# Smart molecular crystal switches

**DOI:** 10.1002/smo.20230031

**Published:** 2024-02-16

**Authors:** Ian Cheng‐Yi Hou, Liang Li, Hongyu Zhang, Panče Naumov

**Affiliations:** ^1^ Smart Materials Lab New York University Abu Dhabi Abu Dhabi United Arab Emirates; ^2^ State Key Laboratory of Supramolecular Structure and Materials College of Chemistry Jilin University Changchun China; ^3^ Center for Smart Engineering Materials New York University Abu Dhabi Abu Dhabi United Arab Emirates; ^4^ Research Center for Environment and Materials Macedonian Academy of Sciences and Arts Skopje Macedonia; ^5^ Department of Chemistry Molecular Design Institute New York University New York USA

**Keywords:** martensitic transition, molecular switch, phase transition, single crystal, smart material

## Abstract

The multifaceted switches are part of our everyday life from the macroscopic to the molecular world. A molecular switch operating in the solution and in the crystalline state is very different. In this review, we summarize the state‐of‐the‐art of smart molecular crystal switches based on molecular martensites. These crystal switches respond to external stimuli and reversibly change between states, retaining their macroscopic integrity. The operation of the switches predominantly relies on temperature alterations or mechanical stress, with emerging methods based on photothermal effects, photoisomerization, and host‐guest chemistry. The capability of changing the molecular orientation and interaction in smart molecular crystal switches offers opportunities in several applications, including actuators, reversibly shaping structural materials, optoelectronic and magnetic materials, as well as switchable porous materials. Smart molecular crystal switches have vast potential in modern scientific and technological progress. The ongoing research shapes a rich landscape for innovation and future scientific exploration across diverse disciplines.

## Graphical abstract

The amplification of the phenomenon of switching from the level of molecule to crystal is not a straightforward exercise due to the restrictive space available in the crystal. However, that brings many benefits such as cooperative phenomena that are related to very fast and efficient switching, and the opportunity to use diffraction methods for the elucidation of the underlying mechanisms. This review aims to summarize the state‐of‐the‐art in the field of crystalline switches within the broader context of smart materials.

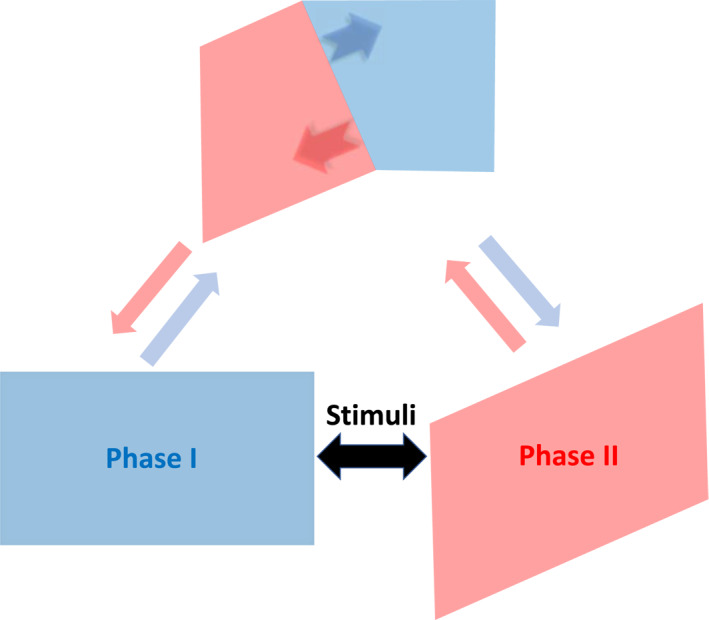

## 1 INTRODUCTION

**FIGURE 1:**
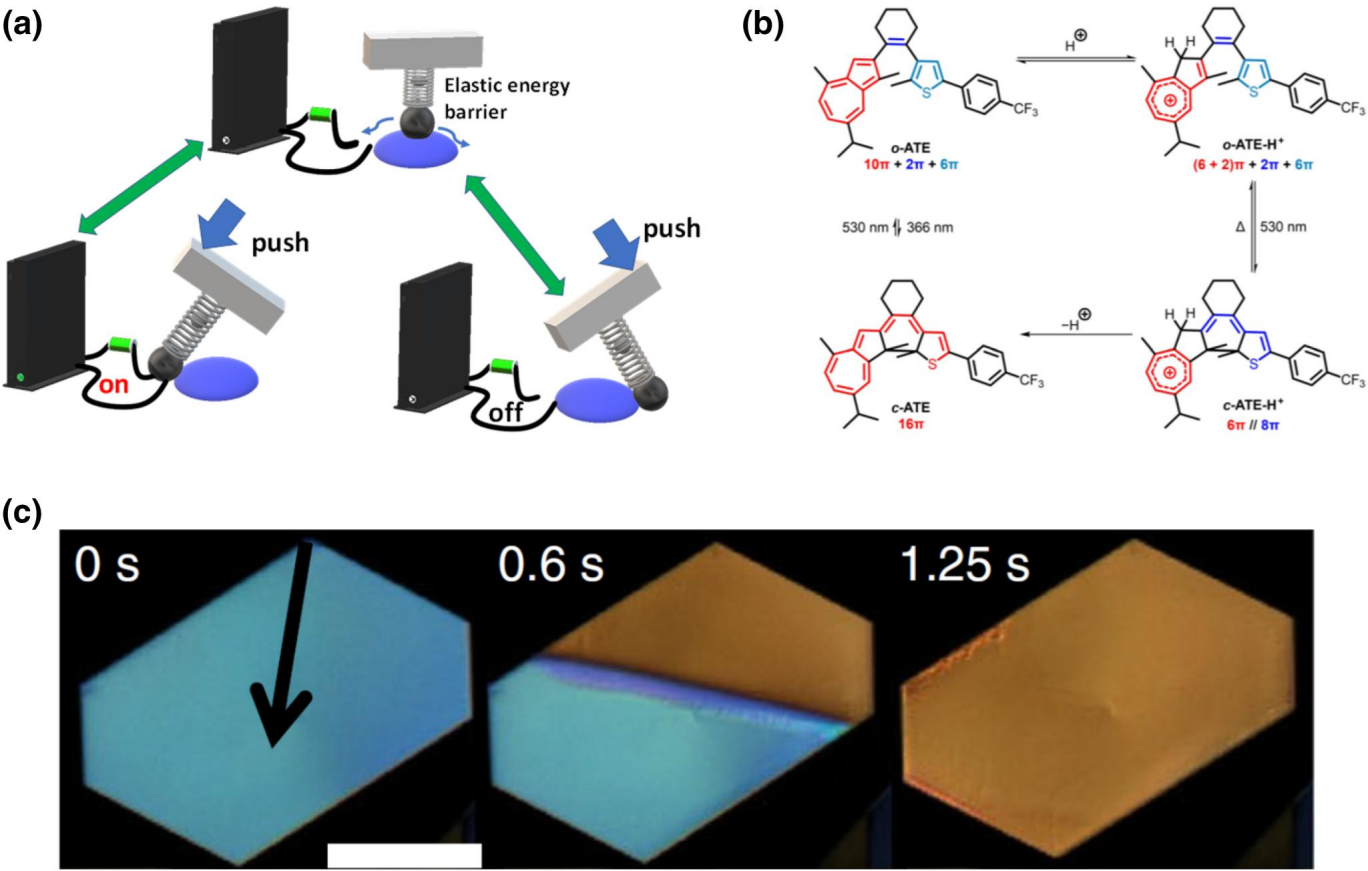
Examples of switching at different levels: device, molecule and molecular crystal. (a) Switching on and off an electronic device by pressure, (b) switching between closed and open forms of a molecule by using a photon and a proton,^[1]^ and (c) switching among different phases of a crystal of an organic martensitic material by changing ambient temperature.^[2]^ Reproduced under the terms of CC‐BY licenses.[^1^, ^2^]

The daily act of switching indoor lighting on and off, driven either directly by human intervention or indirectly, through sensor technology, exemplifies the three fundamental attributes of a switch: (1) a system that can exist in distinct states, (2) application of a specific stimulus to the system beyond a certain threshold leads to a directional, abrupt, and complete transition among the states, and (3) the transition between the states is reversible (Figure 1a). Switches are present in our lives and are used from daily operations such as controlling of light to rover scouting on the surface of Mars. It would not be too much to say that the quest for precise, repeatable control over the switching events is an important component of the societal development.

Aside from the world we can see, both nature and chemists have developed “smart” (macro)molecules that function as switches on a much smaller scale (Figure 1b).[^3^, ^4^] Operating these molecular switches poses different challenges compared to their macroscopic counterparts, and the molecular switches normally rely on reversible physical processes or chemical reactions. Depending on the stimulus that drives the process of switching, they can be categorized as photoswitches, redox‐switches, mechanoswitches, or simply, chemical (especially acid‐base) switches.^[5]^ The photoswitches are arguably the most popular systems due to the rapid interactive and traceless nature of the photon, and the opportunities for achieving high spatial and temporal resolution (Figure 1b), and are continuously inspirin the development of novel photochromic molecular systems.^[6]^ The temperature as an effector is not reliable to induce a switch‐like behavior at a single molecular scale, regardless if it activates a chemical reaction, conformational change, or a formation of a supramolecular interaction. Namely, at the desired timescales of switching, the chemical equilibrium has a low sensitivity to temperature variations, and the equilibration that occurs after the temperature has been changed is a very slow process. The rise of the temperature itself causes more equally populated low‐temperature and high‐temperature states (LTS and HTS, respectively), compromising the free energy difference. For example, a recent report describes a temperature‐dependent emission in a pyrene‐containing homo[2]catenane.^[7]^ As a dispersed molecule in solution, the fluorescence intensity of the catenane was found to drop by more than 60% when heating the sample from 288 to 353 K; yet, this variation in emission is gradual and occurs without a distinct transition point.

Although a molecular switch could operate perfectly at a single molecular level, such as in single molecular junctions,^[8]^ in solution‐based molecular switches the switching behavior smears in most of the working scenarios as a result of the loss of cooperativity between the molecules. Consequently, although each individual molecule operates as a switch, the effect of that change in the collective, macroscopic property becomes slow and continuous. This problem could be complicated further when a chemical equilibrium is established in the solid state.^[9a]^ The solution‐based molecular switches, including the photoswitches, redox‐switches, and chemical switches, rely on the interaction of molecules with photons, electrons, or chemical agents, respectively. These particles are not always able to deeply and effectively penetrate into condensed materials, resulting in a sluggish response and inhomogeneous distribution of the switching events. For example, the photostationary state of the spiropyran derivative single crystal contains a much lower amount of the merocyanide form in comparison to the switching in solution.^[9b,9c]^

In contrast with solution‐based switches, condensed state molecular switches can employ an alternative working mechanism, that is, a phase transition which occurs in an ordered, well‐defined environment (Figure 1c). Phase changes are introduced by field‐based stimuli, such as thermal, electric, magnetic and force fields. These stimuli are able to penetrate deeper into a material and affect more molecules. In contrast to switching in the solution, where molecules gradually redistribute between LTS and HTS in response to a changing field, the molecules in a condensed phase closely interact with each other and local changes at the molecular level can pass on. In liquid crystals, the relatively high freedom of molecular motion provides a basis for amplification of the single molecular switching to a larger scale, allowing sophisticated controlloing of molecular orientation.^[9]^ In contrast, switching molecules in the solid state is more difficult due to the restricted conformational landscape. However, solid‐state materials can react to additional stimuli such as a force field. Furthermore, the long‐range order in molecular crystals offers efficient transduction of stimuli, resulting in autocatalysis/self‐polarization and an abrupt and collective macroscopic switching effect.[^10^, ^11^, ^12^]

Anisotropic orientation of molecules in single crystals offers useful properties such as birefringence, dichroism, pleochroism, polarized emission, piezoelectricity, and polarized waveguiding.[^13^, ^14^, ^15^] These properties change between different states in a molecular single crystal switch, creating materials with versatile applications. Importantly, for a complete switching cycle, that is, after the material returns to the initial state, it is crucial that the single crystal retains its macroscopic integrity through a single‐crystal‐to‐single‐crystal (SCSC) process, especially when the properties related to anisotropic orientation of moelcules are in consideration or when the crystal is applied as (a part of) structural material. The molecules in molecular crystals are glued together by non‐covalent weak interactions, such as hydrogen bonds, halogen bonds, chalcogen bonds, and dispersion forces, rendering such crystals with a generally low mechanical strength. Thus, retaining macroscopic integrity can be challenging for many potentially switchable molecular single crystals. Two major types of molecular single crystalline switches are organometallic complexes exhibiting spin‐crossover or inter/intramolecular charge transfer, and hydrogen‐bonded supramolecular polymers of organic molecules that are capable of proton transfer and proton‐coupled charge transfer.[^14^, ^16^] The chemical transitions involved in these two types of molecular crystals are related to small changes in the molecular conformation. As a result, their crystal lattices are not severely altered during the switching event, leading to small‐induced strain/stresses that secure a retention of the macroscopic integrity of the crystal in an SCSC fashion. However, such small alterations of the molecular skeleton and conformation also limit the switching of molecular orientation‐related properties and functions that requires significant changes of the crystal lattice. For example, for crystalline actuators, the maximum work output is defined by the maximum strain and force that can be induced during a switching event^[17]^; a small induced strain would make an actuator much less powerful.

There is another type of a more “physical” SCSC process, often referred to as molecular martensitic transition based on their similarity to the austenite–martensite transition of steel,^[18]^ that can display more drastic molecular orientational changes while maintaining the single crystal intact. The most important molecular mechanisms involved are conformational and supramolecular interaction changes. These mechanisms are irrelevant for solution‐based molecular switches; however, they are critical for the solid state. Martensitic transitions can be considered as switching phenomena because they start with nucleation of the emerging phase and progress to the transition is thresholded.^[19]^ The emerging phase normally propagates with a clear and flat phase front where the emerging phase can seamlessly “grow” at the interface by converting the neighboring existing phase “preparing” for the transition implying that the transition can propagate ultra‐fast without molecular diffusion (Figure 1c). The coherent and continuous interface between the two changing phases facilitates a highly reversible transition with retention of macroscopic integrity.

In this review, we present the state‐of‐the‐art smart molecular crystal switches mainly based on molecular martensites. Examples are carefully chosen from dynamic molecular crystals that exhibit stimuli‐responsive abrupt, thresholded, and microscopically and macroscopically reversible switching among states. In Section 2, we discuss the general external stimuli used to operate a smart molecular crystal switch and how they normally react to the stimuli. In Section 3, we summarize the sort of property that is switched in these smart molecular crystal switches. We note that there are several excellent recent reviews that address fundamental aspects regarding molecular martensites, including their molecular mechanism, theoretical background, and crystal packing.[^10^, ^19^, ^20^, ^21^] In this review, we will not discuss these details, which are well covered previously.

## 2 OPERATING A SMART MOLECULAR CRYSTAL SWITCH

Change of the ambient temperature and the application of stress to the crystal are the most common approaches to operate switching events in smart molecular crystal switches based on the conformational and supramolecular interaction changes. There are some alternative approaches, such as the use of photothermal effect to heat up a crystal or trigger a transition of an overcooled crystal by impact. Other stimuli can also be used, including electric/magnetic field, guest molecules, and photoreactions; however, they are practically more challenging and also less successful. In the following section, we discuss these one‐by‐one and provide real examples.

### 2.1 Thermoswitches

Due to the inherent characteristics and mechanism of the martensitic transition, a significant amount of free energy is rapidly released during the process. This energy could lead to fracturing of the organic crystal despite the theoretical full reversibility of the transition, and the retention of the macroscopic integrity during the transition cannot be predicted. In extreme cases, the released energy may propel the crystal to jump a distance considerably greater than its actual size, a phenomenon known as “thermosalient effect”.[^20^, ^22^, ^23^] This effect can result in various outcomes, ranging from simple cracking and splitting, to more severe consequences such as explosion or peeling off.^[20]^ Practically, transitioning of the crystal in damping surroundings, such as immersion in oil, proved beneficial in maintaining the crystal integrity during the transition.^[24]^ Aside from the obvious damage from the thermosalient effect, there may also be subtle deterioration of crystal quality that remains visibly undetectable. The transparency of the crystal may progressively decrease after several switching cycles due to the formation of small cracks.^[25]^ Unlike solid‐solid transitions, other phase transitions, such as melting and sublimation, induce irreversible changes to the shape of the crystal. A high sublimation rate^[26]^ or a melting point close to the solid–solid phase transition temperature^[27]^ will similarly impact the effective utilization of a thermally operated smart molecular crystal switch.

**FIGURE 2:**
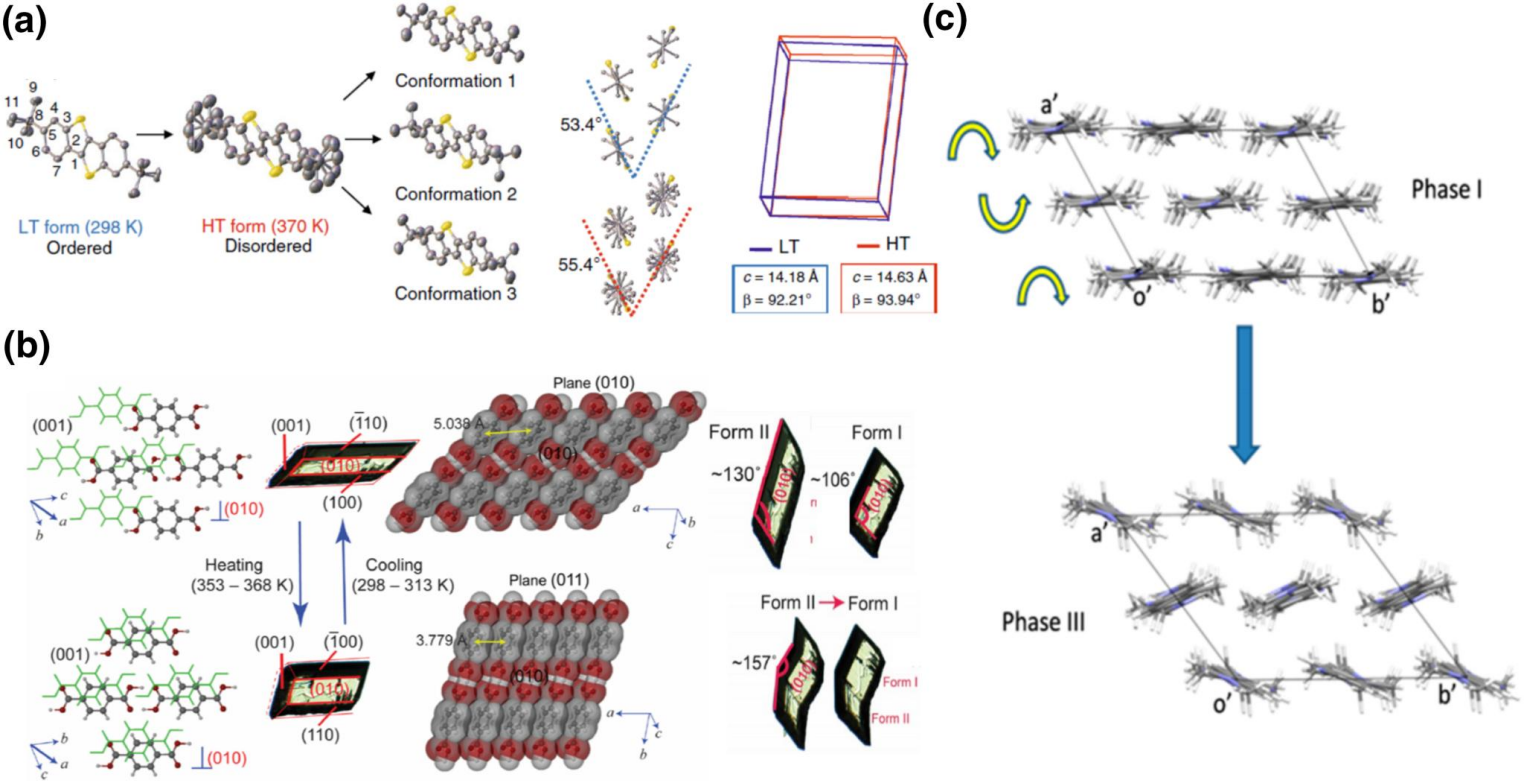
Examples of crystalline thermoswitches. (a) 2,7‐Di‐*t*‐butyl[1]benzothieno[3,2‐b]benzothiophene (DTBTBT),^[2]^ (b) terephthalic acid,^[24]^ and (c) 4‐dimethylaminobenzaldehyde(4‐cyanophenyl‐ethylidene)hydrazine.^[28]^ Reprinted (adapted) with permission from Karothu et al.^[24]^ and Centore et al.^[28]^ Copyright 2016, 2018 American Chemical Society. Reproduced under the terms of the CC‐BY license.^[2]^

The molecular mechanism of a thermally operated smart molecular crystal switch has been discussed in detail in a recent comprehensive review by Park and Diao.^[19]^ The most straightforward and arguably the most predictable mechanism involves an order‐disorder transition of specific functional groups, typically alkyl groups,[^2^, ^28^, ^29^, ^30^, ^31^, ^32^, ^33^] since the phase transition by temperature change is to a large extent controlled by the entropy. This mechanism is associated with amphidynamic stator‐rotor molecular crystals that have a well‐established developmental history.^[34]^ In these crystals, the “stator” components are normally larger moieties with low motility that remain static over a wide temperature range, while the “rotor” part is frozen below a certain temperature and becomes dynamic above a critical temperature, undergoing order‐disorder transitions. The advantage of this type of transition is a minimal disruption to the intermolecular interaction, preventing the development of significant strain during the transition and thereby facilitating the retention of macroscopic integrity. However, due to the limited change in crystal lattice, the property difference between HTS and LTS is generally small. For example, upon heating above 347 K, 2,7‐di‐*t*‐butyl[1]benzothieno[3,2‐b]benzothiophene (DTBTBT) transitions from LTS to HTS where the *t*‐butyl group populates three conformations (Figure 2a).^[2]^ The effect of the crystal lattice is an increase in the length of the crystallographic *c* axis from 14.2 Å to 14.6 Å and angle *β* from 92° to 94°. The overall macroscopic effect is barely observable.

A large alteration of crystal lattice requires molecular mechanisms other than order‐disorder transition. Hydrogen‐bonded structures such as terephthalic acid and guanidinium nitrate form molecular layers by strong intermolecular interactions. The molecular layers are robust and slide over each other when transferring from LTS to HTS (Figure 2b).[^17^, ^24^] Molecules can also become less ordered in a closely packed setting, and this reflects in the changes in crystallographic parameters. For example, 4‐dimethylaminobenzaldehyde(4‐cyanophenyl‐ethylidene)hydrazine transforms from an AA‐stacked LTS to an AB‐stacked HTS with a herringbone‐type stacking, increasing the entropy of the system (Figure 2c).^[28]^ The sizable alteration on the intermolecular interactions also reflects on the size and shape change of the crystal (Figure 2b,c).

High phase‐front propagation speed (concerted transition) is a known characteristic of a martensitic transition. The switching of a smart molecular thermoswitch is normally completed within a second, and occurs in some cases on a millisecond time scale, which is much faster than the propagation of other solid‐state transitions such as those triggered by a spin crossover.^[35a]^ Among the molecular crystalline martensites, the structures that include strong intermolecular interactions are faster compared to those triggered by an order‐disorder transition. For example, the phase front in crystals of the terephthalic acid and pyroglutamic acid can propagate up to 500 mm/s during a transition, which is fast enough to generate detectable acoustic burst.^[35]^ In contrast, the propagation speeds are usually below 1 mm/s in case of side chain order–disorder driven transitions.^[33]^

### 2.2 Mechanoswitches

**FIGURE 3:**
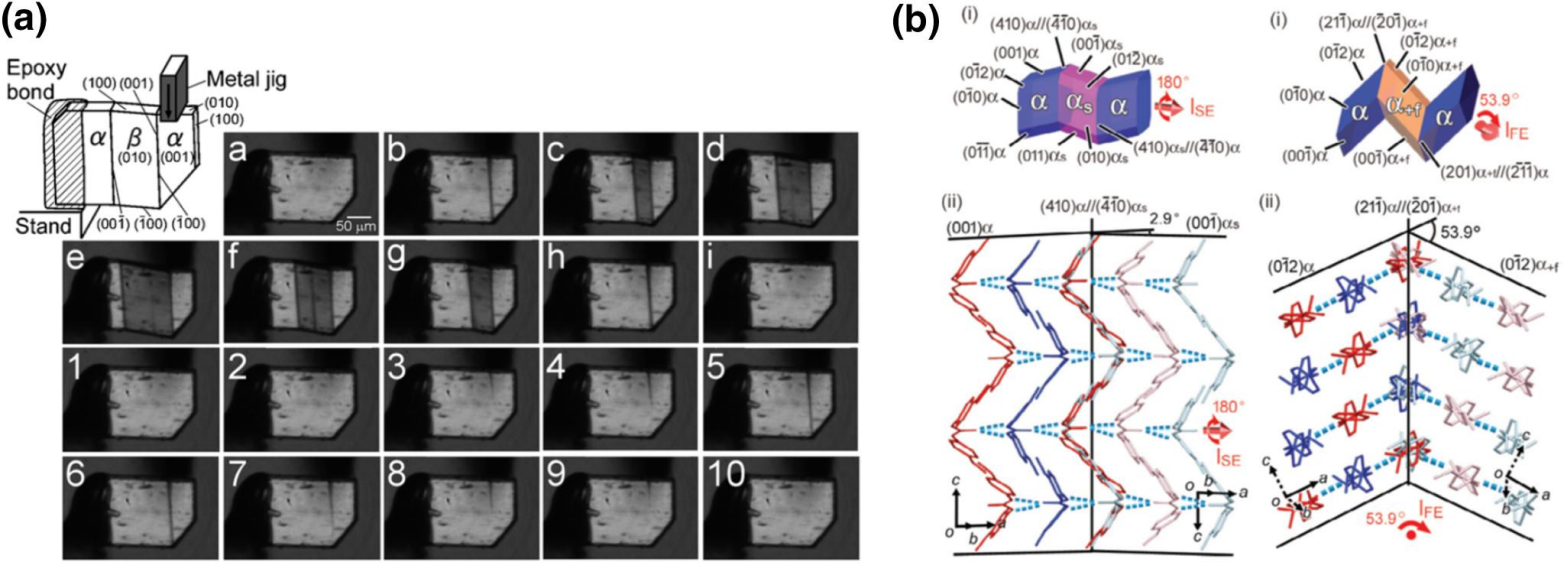
Examples of crystalline mechanoswitches. Shear‐stress induced (a) superelastic (SE) phase transition of terephthalamide^[37]^ and (b) SE (left) and ferroelastic (FE) (right) twinning of *N,N′*‐bis‐(4‐methoxyphenyl)urea.^[38]^ The event sequence in (a) is a → 1 to 5 → b to h → 6 to 10. Reprinted (adapted) with permission from Takamizawa et al.^[37]^ and Sasaki et al.^[38]^ Copyright 2016, 2020 John Wiley and Sons.

The thermally induced transition described above could be triggered by a proper external force applied along a certain crystallographic direction.^[36]^ The net result is a conversion from a thermodynamically more stable state to a less stable one, and the additional free energy is supplied by the mechanical work done by the external force. Because of the induced instability, the less stable state could spontaneously revert back to the more stable state when the external force is reduced. The whole forward, mechanically induced, and reversed, spontaneous phase transition is described as *superelasticity* (SE). It is attributed to a kind of elasticity since the mechanical process is reversible, and the prefix “super‐” alludes to the much higher strain that can be induced compared to a regular elastic crystal due to the involvement of phase transition. In a typical example of an SE transition, when shear stress is applied to single crystal of terephthalamide along the [$\bar{1}$00] direction, the LTS *β* phase nucleates inside the HTS *α* phase near the applied position of the shear force. The emerging *β* phase then propagates with a single and flat advancing phase front until the fixing point. After the shear stress is reduced to the critical value, the induced *β* phase spontaneously transitions back to the thermally more stable *α* phase (Figure 3a).^[37]^

Smart molecular crystal mechanoswitches can also respond to applied force by stress‐induced twinning. The twinned crystals include an interface between the parent component and its twin, and the interface can be generated and moved by a suitable shear force. The free energy change of altering the interface is in principle zero since there is no phase transition involved. During stress‐induced twinning, molecules rearrange while the phase‐front passes through, and the crystal is changed to another orientation (a twinning variant). Because of the energetic scenario, theoretically, when the external stress is removed in a stress‐induced twinning, there is no driving force for the crystal to revert to the original twinning variant. Instead, a shear‐force in the opposite direction is required to reverse the process. This mechanical behavior is described as *ferroelasticity* (FE). The terminology is adopted in analogy with ferroelectricity and ferromagnetism, a self‐polarization phenomenon in which a system can maintain a polarized state even after an external field, which has been used to polarize the system, is reduced or completely removed, and the polarization can be reversed or neutralized with an external field in an opposite direction. In FE, the field is a force field, and the polarization is the spontaneous strain.

Although theoretically SE and FE can only happen in stress‐induced phase transition and twinning, respectively, it has been shown that smart molecular crystals often exhibit stress‐induced SE transition based on twinning,^[^ ^38^, ^39^ ^]^ and SE by twinning could transform into FE when stress is applied over longer periods of time.[^40^, ^41^, ^42^] For example, stress‐induced transformation of a smart molecular crystal mechanoswitch based on *N,N′*‐bis‐(4‐methoxyphenyl)urea can be triggered by application of a shear stress along the [001] and [0 $\;\bar{1}$2] directions, leading to SE and FE behavior, respectively (Figure 3b).^[38]^ Though with very different strains, both transitions are based on twinning. Notably, the stress‐induced phase transition can be “frozen” if the chemical driving force for the reverse transition is small, and the system could exhibit a pseudo‐ferroelastic behavior.^[^ ^43^, ^44^, ^45^ ^]^

### 2.3 Potential usage of other stimuli

Stimuli other than changing the ambient temperature or shear stress have also been used to induce property changes in molecular crystals, albeit usually not as a switching process. Photoirradiation is one of the most favored means to switch the structure owing to a number of advantages it has over other forms of stimulation. The effects can be physical, such as photothermal transition that heats up molecular crystals or chemicals, such as photodimerization of molecules therein. The macroscopic response of molecular crystals upon light irradiation is diverse. Photothermal effect or photochemical reactions can induce bending of molecular crystal by uneven thermal expansion or distribution of photoproducts, respectively, which cause stress gradient within the crystal lattice that may even lead to sudden explosion of the crystal (photosalient effect).[^20^, ^22^, ^23^] However, most of these dynamic behaviors cannot be considered as switching because of lacking macroscopic or microscopic reversibility, distinct states, and a clear transition threshold.

The most straightforward photophysical way to operate a smart molecular crystal thermoswitch is by using the photothermal effect to induce its phase transition. In principle, most of the smart molecular crystal switches can be triggered photothermally due to the non‐radiative relaxation pathways in the solid state. One example is a compound reported by Horie et al. They demonstrated that the transition temperature of the LTS to HTS transition of a ferrocene salt decreases 40 K when the compound is irradiated at 445 nm with a 20 mW laser, and the transition completes within 25 ms from the onset.^[46]^

Molecular photoswitches are at the core of the smart molecular crystals since they involve a photochemical reaction that significantly alters the molecular electronic structure. Such change usually renders much larger difference between the two states compared to a conventional mechanoswitch or a thermoswitch based on changes in conformation or supramolecular interactions. However, realizing the switching behavior in the crystalline state poses additional challenges. Firstly, light cannot penetrate deep inside the crystal since (some of) the photoproducts generally absorb light at the same wavelength. Secondly, with high light intensity, even if the photon density is uniform inside the crystal, the molecules are not able to attain a complete conversion due to the establishment of a photostationary state(s). Thirdly, many photoswitches undergo large geometrical changes between isomers. The tightly packed crystal lattice is usually prohibitive for such transformation. Because of these reasons, photoswitches generally react slowly and have very low conversion in the crystalline state.[^47^, ^48^, ^49^] Although the small amount of the photogenerated form can still induce local stress and macroscopically observable change in the crystal shape, the changes are gradually, which disqualifies these crystals as macroscopic switches where an abrupt transition between the states is required.

**FIGURE 4:**
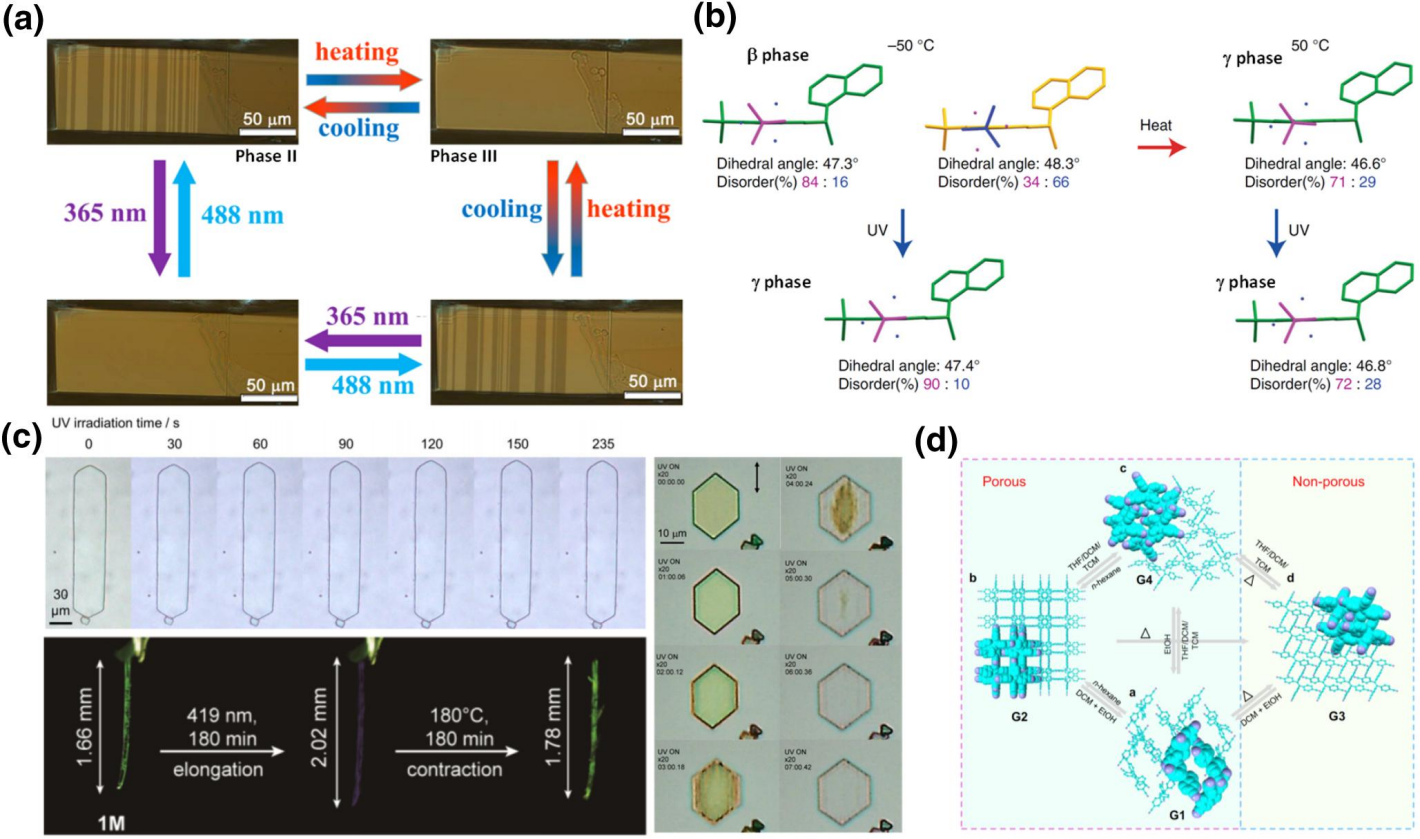
Potential crystalline photo switches and chemical switches. Photoinduced (a) twinning/detwinning^[50]^ and (b) phase transition^[33]^ of salicylideneaniline compounds and (c) shape and color change during the photoreaction of several negative photochromic compounds in the crystalline state.[^51^, ^52^, ^53^] (d) Changing porosity of a molecular crystal by guest inclusion.^[54]^ Reprinted (adapted) with permission from Liao et al.^[50]^ and Chen et al.,^[51]^ copyright 2021, 2023 American Chemical Society. Reprinted (adapted) with permission from Morimoto et al.,[^52^, ^53^] copyright 2022 John Wiley and Sons. Reproduced under terms of the CC‐BY license.[^33^, ^54^]

Recent reports have shown that partial isomerization of some molecular crystals of salicylideneanilines leads to phase transition of the unreacted molecules. These compounds can undergo photoisomerization between the enol and keto forms. The group of Xiong reported a smart molecular crystal thermoswitch based on a *tert*‐butyl‐substituted chiral salicylideneaniline.^[50]^ Since the HTS phase III is paraelastic and the LTS phase II is FE, cooling from phase III to phase II leads to spontaneous twinning (Figure 4a). Interestingly, the twinning/detwinning can also be induced by irradiation at 488 and 360 nm, respectively. The authors attribute the photoresponse as a direct result of the photoisomerization between the enol and keto forms of salicylimine. The group of Koshima reported earlier a smart molecule thermoswitch based on another structurally similar salicylidineaniline derivative, which undergoes transition between LTS *β* phase and HTS *γ* phase at around 313K. Remarkably, when irradiated at 365 nm, LTS *β* phase already transitions to HTS *γ* phase at 223 K (Figure 4b).^[33]^ The reverse phase transition from the photoinduced *γ* phase happens at a time scale comparable to the thermal half‐life of the keto‐forms generated by irradiation, suggesting that the photoisomerization plays a significant role in the anomalous phase transition.

Negative photochromic compounds can be a viable choice for the development of smart molecular crystal photoswitches. In a negative photochromism, the photoproduct absorbs at a wavelength shorter than the reactant, facilitating both deep penetration of light into the crystal and a complete photoconversion. Photoinduced pericyclic reactions are often negative photochromic since they generally result in photoproducts with more isolated conjugated systems. Very recently, 100% conversion has been demonstrated in rod‐like^[51]^ and plate‐like[^52^, ^53^] crystals by utilizing pericyclic reactions (Figure 4c), although the reactions are often found to proceed gradually and the reverse reactions require heating to elevated temperatures (Figure 4c, left).[^51^, ^53^] The high yield of the photoreaction can have a prominent effect on the crystal shape, where crystals can elongated to 7% (plates) and 22% (rods) (Figure 4c, left). Notably, a cooperative photodimerization has been recently observed with 2,5‐distyrylpyrazine single crystal (Figure 4c, right).^[52]^ After irradiating the crystal for around 2 minutes, it suddenly reacts and the whole crystal is transformed quickly. Such a thresholded reaction is the criteria for a switch‐like behavior.

Host–guest interaction provides another opportunity for the development of new types of smart molecular crystal switches. It has been shown in several molecular crystal systems that losing/gaining cocrystalizing components can lead to interesting stimuli responsive behaviors[^55^, ^56^, ^57^] sometimes even Martensitic‐transition‐like.^[58]^ The recent development of dynamic intrinsic porous^[59]^ or framework[^60^, ^61^, ^62^] molecular crystals carries the potential for the design of smart crystalline chemical switches. The crystal structures of these porous molecular crystals can be reversibly “tuned” by incorporating different guests, and the molecular conformation of the host is altered accordingly. This is particularly interesting when some of the properties of the host are sensitive to the molecular conformation, as it has been shown for the dynamic single crystal of aggregation‐induced emissive tetraphenylethene derivatives (Figure 4d).[^54^, ^63^]

## 3 PROPERTIES OF MOLECULAR CRYSTALS SWITCHES

The main purpose behind the design of switches is its functionality, and the function emerges from the different properties of the different states. In this sense, it can be argued that a smart molecular crystalline switch involving a phase transition would be practically more useful than that based on twinning. Namely, the phases of a molecular crystal intrinsically have different properties that stem from difference in molecular conformation and/or supramolecular interaction. The most straightforward function can be directly related to the macroscopic change of the shape of the crystal during the phase transition, such as reorienting or connecting/disconnecting an object. Also, reshaping of the crystal, powered by the decrease in chemical‐free energy, can be applied to do mechanical work analogous to actuation. Several other properties related to the intermolecular interactions could also change between phases, for example, light absorption/emission, charge carrier mobility, proton conductivity, etc.

The anisotropy in the orientation of the molecules in molecular crystals offers an opportunity for the crystal to interact with external stimuli in different ways, depending on the crystal orientation. Thus, although the change in the molecular orientation within a crystal would not necessarily alter the absolute macroscopic properties, it could vary the directionality of, for example, voids and channels, charge carrier/proton conduction, (transition)dipolar/magnetic polarization direction, etc. Both a phase transition and a stress‐induced twinning can change the molecular orientation. However, twinning generally can reach a larger angle of molecular rotation since the applied stress can overcome the larger corresponding frictional force. Also, twinning only changes the anisotropicity of the crystal but not the absolute property. In certain circumstances, from a practical perspective, the stress‐induced twinning can thus be more useful than a phase transition. In the following sections, we describe the switching of several classes of properties in smart molecular crystal switches.

### 3.1 Actuators

**FIGURE 5:**
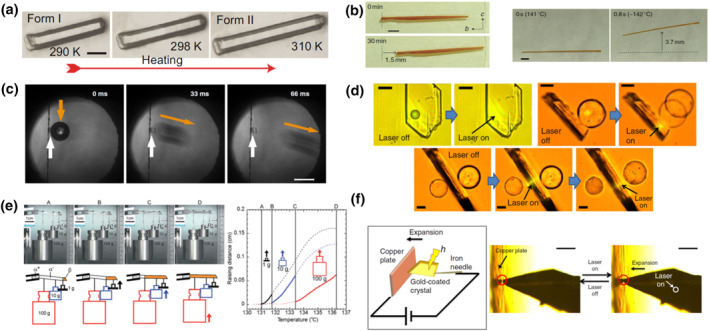
Smart molecular crystal thermoswitches operating as reusable actuators. (a) Colossal elongation of a good‐quality crystal of guanidinium nitrate,^[17]^ (b) self‐propulsion of a single crystal of an azobenzene derivative,^[32]^ (c) actuation based on a single crystal of 9‐oxofluorene derivative,^[64]^ (d), (f) spatially‐controlled actuation and on‐off switching of electric circuit based on a laser‐triggered phase transition of a ferrocene salt crystal,^[46]^ (e) weight lifting based on SE and FE behavior of tetra‐*n*‐butylphosphonium tetraphenylborate single crystal.^[65]^ Reproduced under terms of CC‐BY licenses.[^17^, ^32^, ^46^, ^64^, ^65^]

The actuators made of molecular crystal thermoswitches have several advantages over actuators based on other materials. The group of Naumov has highlighted that molecular crystal thermoswitches are beneficial for being low‐density materials because they are normally purely organic.^[35a]^ They compared organic actuating crystals to other actuators and concluded that the rigid and martensitic nature of the smart molecular crystals turns them into ultra‐fast switching actuators with high power density.[^17^, ^35^] This is also because during the phase transition, molecular martensites can suddenly expand, in some cases even nearly half their length (Figure 5a).^[17]^ Other processes, such as thermal expansion, are slower and generally in the range of 0−20 × 10^−4^% K^−1^ with the largest cases reaching 3.6 × 10^−2^% K^−1^.[^66^, ^67^]

The most straightforward mode of actuation is self‐propulsion, where a change in shape induced by a phase transition of smart molecular crystal thermoswitches exerts force onto the substrate, which propels that crystal. The thermosalient effect can be useful for actuation by this mechanism, although it is normally destructive. The group of Naumov has discovered that hydrogen‐bonding smart molecular crystals of pyroglutamic acid display a particularly robust thermosalient effect.^[67]^ The crystals can jump during at least 15 cycles of phase transition without breaking. The robustness of the crystals of pyroglutamic acid also qualifies them as a coating material for a thermal control over the natural vibration frequency of a cantilever as part of a microelectromechanical system.^[68]^ Unlike the thermosalient actuation, a non‐thermosalient actuation may exert a smaller force on the substrate, and yet it could be more suitable to realize directional movement. The group of Koshima demonstrated robotics‐like motion of crystals of azobenzene derivatives in several cycles of phase transition (Figure 5b).^[32]^ The crystal elongates roughly along its long side for 4.5% when going from LTS to HTS, and this is accompanied by bending of the crystal during the transition. A wide crystalline rod can directionally “crawl” for about 18% of its length over 30 min of repetitive phase transition cycles (Figure 5b, left). On the other hand, a narrower crystalline rod bends much more during the phase transition, and can thus “roll” on its side at a much faster rate (Figure 5b, right).

Smart molecular crystal thermoswitches can also be employed to work on other objects. The groups of Xu and Rasing demonstrated an actuator based on the exceptionally robust thermoswitch made of a 9‐oxofluorene derivative.^[64]^ Upon the LTS to HTS transition, a crystal with a size of 200 × 200 × 50 μm^3^ can kick‐off a glass bead (0.15 mg), causing it to roll several centimeters away (Figure 5c). The work density is estimated to be as high as 270 J/kg. A similar application is demonstrated by the group of Naumov for the molecular martensite hexamethylbenzene, although the material has a low sublimation temperature and thermal robustness could be important for prolonged applications.^[26]^

Photothermally induced thermoswitching allows for a remote, noncontact actuation event.^[46]^ The group of Horie showed that a silicon microparticle placed on the surface of a smart molecular crystal of a ferrocene salt immediately pops up as the crystal is irradiated by a 445 nm laser focused at the crystal body just beneath the silicon particle (Figure 5d). Similarly, the crystal can also push away the particle when it is placed next to it (Figure 5d). Furthermore, when two particles are placed at opposite surfaces of the crystal, the actuation events can be selectively triggered by focusing the laser on the areas contacting the particles (Figure 5d). The maximum weight the crystal can lift is 1650 times larger than the crystal weight, thereby outperforming other crystalline actuators based on photoinduced bending of molecular photoswitches.^[46]^

Combining the FE and SE behavior of molecular martensites can present interesting opportunities for actuation. Takamizawa and Takasaki have investigated such possibility for tetra‐*n*‐butylphosphonium tetraphenylborate (TPTB), which possess an LTS *α* phase exhibiting FE property, and an HTS *β* phase having SE behavior stemming from *α*‐to‐β phase transition, but no FE behavior.^[65]^ When SUS weights are hung on a rod‐like crystal with a 3.8 cm length and 0.18 cm^2^ cross‐section at room temperature, the crystal is bent by a stress‐induced twinning (Figure 5e, left). After heating the bent crystal over its phase transition temperature, it can lift the SUS weights up, since transition from the LTS *α* phase to HTS *β* phase causes detwinning of the crystal (Figure 5e, left). It is interesting to note that the phase transition temperatures under such stressed circumstances are 6−8 K higher than those under stress‐free conditions, depending on the weights applied (Figure 5e, right).^[65]^ This ability stems from the higher chemical driving force required for the nucleation and propagation of HTS to mitigate the negative work done on the crystal during the transition.

Smart molecular crystal actuators can be employed to switch an electronic circuit on and off. Yao ’s group designed a circuit incorporating a single crystal of imidazolium tetrabromocubrate.^[69]^ When the molecular crystal thermoswitch transitions from its LTS to HTS, it elongates and pushes a copper foil toward the electrode to make contact. Upon cooling, the crystal shrinks and the connection is broken. The group of Horie also demonstrated on‐off switching of an electronic circuit by a photothermally triggered phase transition of a ferrocene salt.^[46]^ A crystal of the ferrocene salt was coated with gold to make it electronically conductive, and then integrated into a circuit. By light irradiation, the gold‐coated crystal elongates and contacts a copper plate and conducts electric current (Figure 5f). The process is fast and controlled remotely by taking advantage of the swift photothermal heating. Note that not all the smart molecular crystals elongate upon transition from LTS to HTS, for example, a crystal of a naphthalene diimide derivative shrinks more than 10% upon heating.^[70]^ Recently, the groups of Mendoza‐Cortes and Gassensmith utilized this unique shrinkage upon heating to create an electronic circuit that breaks upon heating.^[70]^ It is worth mentioning that the phase transition of the crystal starts from 316 k, which is conveniently close to room temperature.

### 3.2 Versatile reshaping material

**FIGURE 6:**
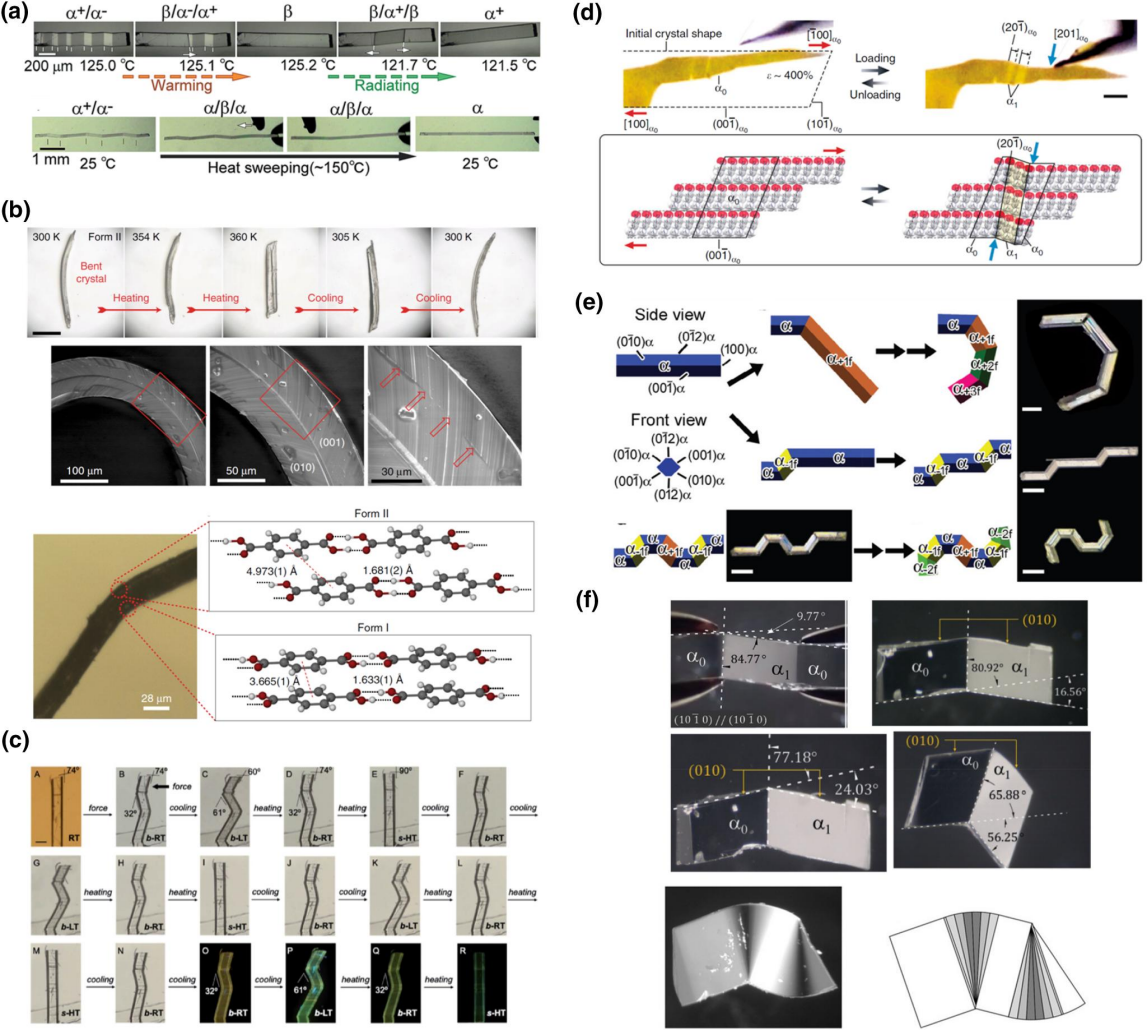
Versatile reshaping smart molecular crystal mechano‐ and thermoswitches. (a) Recovery of the straight shape of tetra‐*n*‐butylphosphonium tetraphenylborate crystal using phase‐transition‐induced detwinning,^[65]^ (b) structure‐straightening of plastically deformed single crystal of terephthalic acid^[71]^ and (c) a gold complex^[72]^ at HTS, (d) stress‐induced SE twinning of a plastically deformed crystal of *p*‐dimethylaminonitrobenzene,^[73]^ and stress‐induced twinning of crystal of (e) *N,N′*‐bis‐(4‐methoxyphenyl)urea,^[38]^ and (f) *p*‐diethoxybenzene.^[74]^ Reprinted (adapted) with permission from Sasaki et al.^[38]^ and Engel et al.,^[74]^ copyright 2020, 2018 John Wiley and Sons. Reproduced under terms of the CC‐BY licenses.[^65^, ^71^, ^72^, ^73^]

Smart molecular crystal switches operating under multiple thermal and mechanical switching modes can manifest high deformability in the generally considered robust crystals. One particular interesting topic is the correlation with shape memory alloy, which stems from the Martensitic transition. The actuation mechanism shown for TPTB is in principle the “recalling” step in the shape memory effect, where the crystal is reversed to its shape memorized during crystallization.^[65]^ For a zig‐zag‐shaped TPTB crystal (deformed by stress‐induced twinning at its LTS FE *α* phase) shown in Figure 6a, heating over α→β phase transition temperature causes the crystal to detwin. The consecutive cooling from the HTS *β* phase back to LTS *α* phase converts the crystal into the “original” single‐domain crystal before stress‐induced deformation. Practically, the shape‐recovery process can be completed by sweeping a hot tip over the deformed crystal from one side to another (Figure 6a). Another similar example is the crystal of pimelic acid.^[75]^ Pimelic acid exhibits LTS phase III and an HTS phase II. Phase III→phase II transition can be induced by stress applied along [101] in an SE way.^[44]^ Interestingly, when the applied stress is held for 3 s, the formed HTS phase II does not spontaneously reverse to LTS phase III anymore, and remains supercooled at room temperature (pseudo‐FE). However, when the temperature is abruptly decreased from room temperature to 213 K, the high chemical driving force causes the supercooled phase II to revert back to LTS phase III, thereby completing the shape‐recovery process.^[75]^ Note that phase III can also be deformed by shear‐stress‐induced twinning along [001].^[44]^ However, it cannot be reverted to the undeformed original shape in a similar way as TPTB does.

Several other cases exhibit plastically deformed crystals with pseudo‐shape‐recalling at elevated temperature. The group of Naumov reported that a plastically deformed crystal of terephthalic acid changes to a straight shape at high temperature by thermally induced LTS phase II to HTS phase I transition (Figure 6b, top).^[71]^ It is revealed by synchrotron X‐ray diffraction that the stress‐induced plastic deformation is a result of coexisting domains of HTS phase I and LTS phase II at the concave and convex sides of the bent crystal, respectively (Figure 6b, middle and bottom). During the thermally induced phase transition, the conversion of the domain of LTS phase II to HTS phase I causes the crystal to straighten, while the reverse transition after cooling results in the “original” plastically bent shape. Recently, the groups of Seki, Takamizawa, and Ito reported that a single crystal of a gold complex presents a similar phenomenon.^[72]^ The crystal is a three‐stage molecular thermoswitch that possesses a low‐temperature LT state, a room‐temperature RT state, and a high‐temperature HT state with similar crystallographic parameters but decreasing *β* angle of 120.6°, 106.6°, and 90°, respectively. Furthermore, the RT phase undergoes stress‐induced twinning deformation along the [100] direction. When heated over the RT to HT phase transition temperature, the deformed crystal straightens due to transition to the more symmetric orthorhombic space group (Figure 6c). After converting of from the HT phase back to the RT phase by cooling, the crystal does not keep the straight shape; instead, the crystal reverses back to the “zig‐zag” shape of the twinned crystal (Figure 6c). Similar behavior was also observed for the RT‐LT transition, and the processes can be repeated over several cycles (Figure 6c).

Some smart molecular mechanoswitches developed by Takamizawa ’s group exhibit exceptionally versatile deformability. The molecular crystal *p*‐dimethylaminonitrobenzene has a crystal structure composed of molecular layers held together by dipole‐dipole and π–π interactions (Figure 6d).^[73]^ The layers are bound by hydrogen bonds N=O⋅⋅⋅H–C, which are weaker compared to, for example, the hydrogen bonds between the carboxylic acid groups in the terephthalic acid. When shear stress is applied along the [00 $\;\bar{1}$] direction, the single crystal of *p*‐dimethylaminonitrobenzene undergoes stress‐induced twinning deformation. In contrast, application of stress along the [100] or [$\bar{1}$00] directions, which are parallel to the molecular layers, results in plastic deformation. The crystal can withstand up to 500% strain without breaking. Interestingly, the deformed crystal, although with a very irregular shape, is still a single crystal, and a stress applied along [00$\bar{1}$] still induces twinning (Figure 6d). When symmetry allows, the twinning can be induced by the application of shear stress onto opposite crystallographic faces. This is the case for molecular crystals composed of *p*‐diethoxybenzene and *N,N′*‐bis‐(4‐methoxyphenyl)urea.[^38^, ^74^] Consequently, unlike most of the twinning‐based smart molecular mechanoswitches that can only be deformed in a zig‐zag shape by continuously applying shear stress to the same crystallographic face of every new phase, such as those shown for TPTB in Figure 6a, molecular crystals of *p*‐diethoxybenzene and *N,N′*‐*bis*(4‐methoxyphenyl)urea can be also bent into a “C‐shape” or an “armchair‐shape” (Figure 6e). Furthermore, the crystal of *p*‐diethoxybenzene exhibits multiple stress‐induced twinning modes that generate interfaces (X $\bar{1}$ 0).^[74]^ These modes can be activated at the same time to achieve a deformed crystal with multiple twinning domains and a pseudo‐round corner (Figure 6f).

### 3.3 Switching light absorption, emission, and light‐matter interaction

Intermolecular interactions can affect the ground state and excited‐state electronic structure, thereby influencing the absorption and emission spectra of the material. Compared to the ground state, the excited state can be more sensitive to the environment. Several processes, such as excimer formation, intersystem crossing, and photoinduced isomerization or proton transfer could all occur in the excited state. Considering the relatively subtle change in conformation as well as intermolecular interaction during state switching of smart molecular crystals, a larger change is expected in emission than in absorption.

**FIGURE 7:**
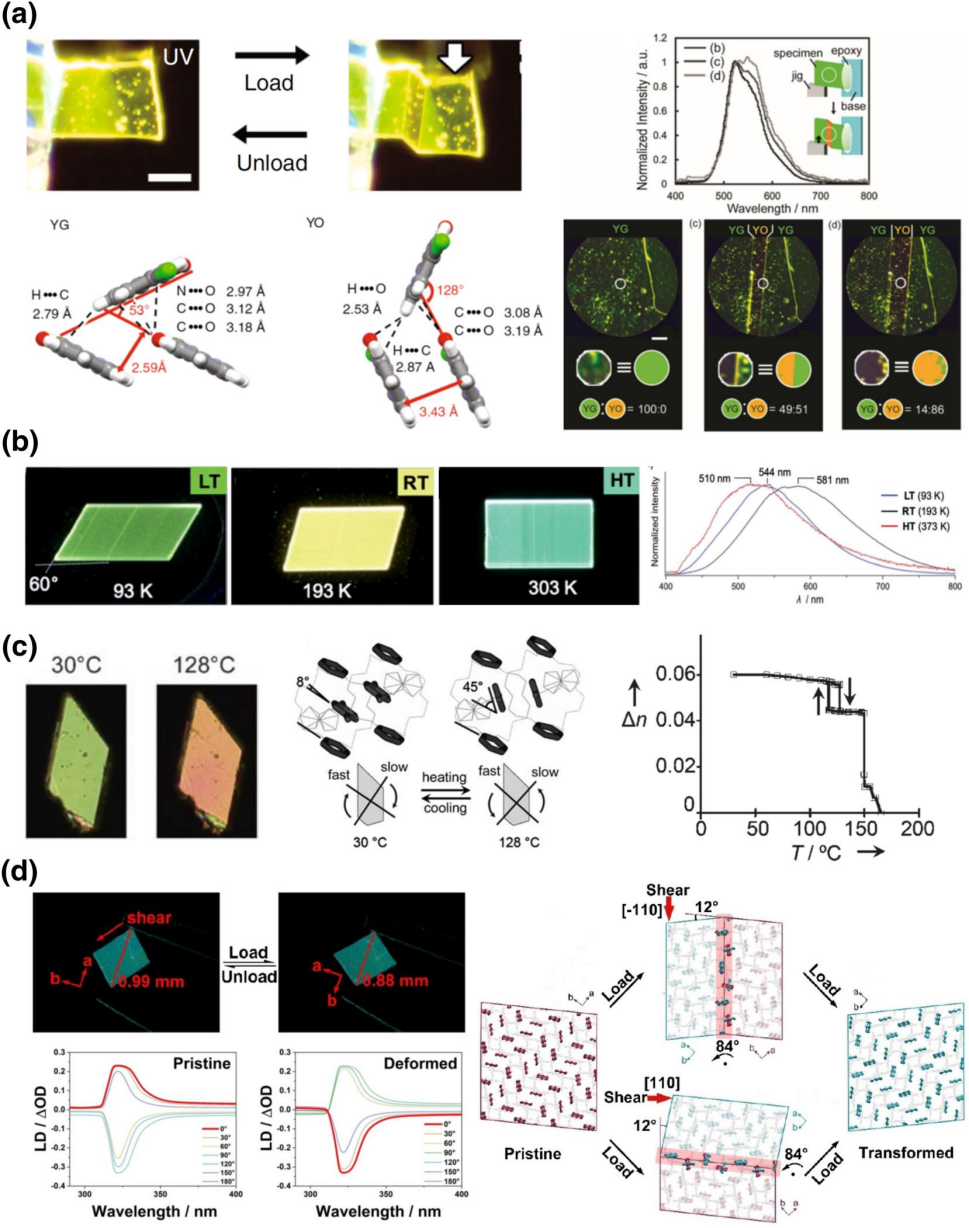
Optical property change of smart molecular crystals. (a) Crystal mechanoswitch of 2‐(imidazo[1,2‐*a*]pyridin‐2‐yl)phenol^[45d]^ and (b) thermoswitch of a gold complex.^[72]^ (c) Interference color and birefringence change upon the phase transition of a ferrocene salt.^[76]^ (d) Switching of light polarized direction by stress‐induced twinning of single crystal of 4,4′‐bipyridinium 3,5‐dimethylbenzoate.^[42]^ Reprint (adapted) with permission from Horie et al.^[76]^ and Li et al.,^[42]^ copyright 2007, 2023 John Wiley and Sons. Reproduced under terms of the CC‐BY licenses.[^45^, ^72^]

Molecular crystals can exhibit distinct emission among different polymorphs. For example, a single crystal of *α*‐cyanostilbene derivative is reported to change its crystal structure and fluorescence color powered by photothermal effect.^[45a]^ However, there are only a limited number of emissive smart molecular crystal switches, and even less with switchable emission properties.^[45]^ The groups of Mutai and Takamizawa reported that the molecular crystalline mechanoswitch 2‐(imidazo[1,2‐*a*]pyridin‐2‐yl)phenol brightly emits through a mechanism that undergoes an excited‐state intramolecular proton transfer (ESIPT).^[45d]^ When shear stress is applied along the [$\bar{1}$ 10] direction of the LTS YG phase, the HTS YO phase nucleates and propagates inside the YG phase in a SE manner (Figure 7a). Both phases are fluorescent through ESIPT with different emission spectra, and the difference in the light can be distinguished by a naked eye. The YO phase exhibits stronger π–π interaction in a J‐aggregate fashion, which can be the reason for the bathochromic shifting emission spectrum (Figure 7a). Note that the stress‐induced YO phase contains certain amounts of YG phase, suggesting that there can be some unconverted domains during the stress‐induced transition (Figure 7a). The groups of Seki, Takamizawa, and Ito reported a molecular thermoswitch based on a perfluorobromophenyl‐substituted gold complex that displays phosphorescence in all of its three phases (Figure 7b).^[72]^ The phosphorescence intensity generally increases upon cooling in all three states. Furthermore, the emission color of the three states can be distinguished visually. The peaks in the phosphorescence spectra undergo bathochromic shifts when the material undergoes a phase transition from the RT phase to both LT and HT phases, which have approximately the same energy gap (Figure 7b). The phosphorescence of all three states was also observed in the stress‐induced twinned crystal (Figure 6c).

In molecules, π‐electrons are often conjugated as a two‐dimensional, planar *π*‐system. Such intrinsic planarity implicates high sensitivity to the polarizing direction of a linearly polarized light. For this reason, molecular crystals can process properties such as birefringence (refraction‐based), and dichlorism and pleochroism (absorption‐based). Crystals having birefringence can exhibit interference color under polarized optical microscope. During a phase transition, the change in the orientation of the molecules also alters the interference color, as demonstrated by the group of Diao, and independently, by the groups of Horie and Osakada in the studies of DTBTBT and ferrocene salt, respectively (Figures 1c and 7c).[^2^, ^76^] Highly anisotropically aligned molecules lead to strong birefringence. A ferrocene salt studied by the groups of Horie and Osakada is one of the cases where all of the phenyl and phenylene groups are aligned in LTS while the phenyl groups are rotated by 37° when entering HTS (Figure 7c).[^76^, ^77^] This results in rotation of the optical axis and weakening of the birefringence during the phase transition (Figure 7c).

The anisotropic interaction with polarized light offers a great opportunity for application based on stress‐induced twinning. Very recently, the group of Yao demonstrated stress‐induced twinning of hydrogen‐bonding cocrystal of 4,4′‐bipyridinium 3,5‐dimethylbenzoate.^[42]^ By applying force along the diagonal corners of this crystal mechanoswitch, the crystal can be completely transformed between two twinning variants (Figure 7d). The net result is the switching of crystallographic *a* and *b* axes of the mother and daughter phases by rotating half of the pyridinyl groups by 90°. This also results in 90° rotation of the direction of polarization plane of the light passing through.

### 3.4 Switching electronic and magnetic properties

**FIGURE 8:**
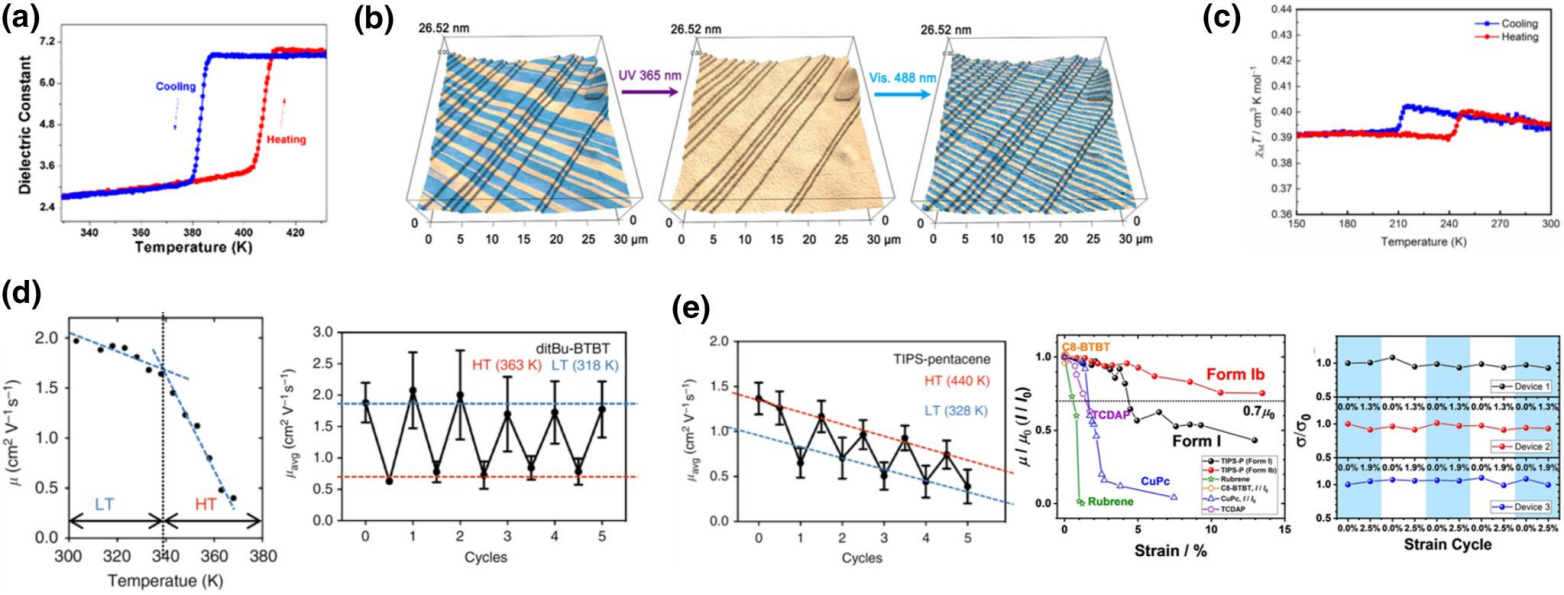
Electronic and magnetic property switching in smart molecule crystals. (a) Switching of the dielectric constant of a crystalline thermoswitch made of ferrocenium tetrachloroferrate.^[79]^ (b) On/off switching of the spontaneous polarization of a salicylidienaniline derivative by photoisomerization.^[50]^ (c) Switching of the magnetic susceptibility of a crystalline thermoswitch of imidazolium tetrabromocuprate.^[69]^ Switching of charge carrier mobilities in smart molecular crystal (d) thermoswitch DTBTBT^[2]^ and (e) thermo/mechanoswitch TMSEP.[^2^, ^29^] Reprinted (adapted) with permission from Zhang et al.^[79]^ and Liao et al.,^[50]^ copyright 2020, 2021 American Chemical Society. Reprinted (adapted) with permission from Wang et al.^[69]^ and Park et al.,^[29]^ copyright 2020 John Wiley and Sons. Reproduced under terms of the CC‐BY license.^[2]^

Phase transition of molecular crystals also affects the dipole and magnetic moment as well as the polarizability in the crystal, thereby altering their intrinsic electronic and magnetic properties. For instance, when heated from 280 to 370 K, the dielectric constant of a single crystal of an azobenzene macrocycle shows a discontinuous increase of around 10% due to a phase transition, and the transition is reversible on cooling.^[78]^ Ionic materials have larger dipole moment and could have larger change in the dielectric constant during the phase transition. Remarkably, the dielectric constant of a ferrocenium tetrachloroferrate single crystal increases more than 100% when transitioning from LTS to HTS, and the value can be reversed to the original value after cooling the sample back to LTS (Figure 8a).^[79]^ As another extraordinary example, the dielectric constants of the HTS and LTS phases of guanidinium nitrate measured separately at room temperature differ for about 30%.^[17]^ Notably, as the group of Naumov has pointed out, the defects formed during the phase transition can have a significant effect on the conductance of this material. Guanidinium nitrate is also a ferroelectric material in which the hysteresis voltage is different for its HTS and LTS phases measured separately.^[17]^ Another, especially interesting ferroelectric smart crystalline thermoswitch is the salicylideneaniline derivative reported by Xiong et al.^[50]^ They reported that the irradiation of the LTS phase II at 365 nm not only results in detwinning of the crystal (Figure 4a), but also converts multiple ferroelectric domains into one (Figure 8b). By further irradiation with a 488 nm light, the spontaneous polarized ferroelectric domains reappear (Figure 8b, right). The authors have attributed this abnormal phenomenon to the increase in the dipole moment from the enol form (3.2 D) to the keto form (4.6 D) of the salicylideneaniline derivative. This material also exhibits reversible alteration of the second harmonic generation between different states as well as a low acoustic impedance.

Several crystalline switches containing magnetic heavy metals whose magnetic susceptibility can change during phase transition were also reported.[^30^, ^69^, ^79^] Compared to other, slower processes, such as spin crossover, that directly change the spin state of metal, the phase transition in the switches discussed here can only alter the degree of quenching between the orbital angular momentum of the ligands surrounding the metal. Consequently, the magnetic susceptibility change is generally small. For example, the magnetic susceptibility remains almost unchanged during the phase transition of ferrocenium tetrachloroferrate.^[79]^ However, around 3% change in magnetic susceptibility was measured during the phase transition of a cobalt complex and imidazolium tetrabromocuprate (Figure 8c).[^30^, ^69^]

Switching of the molecular crystals can also alter their charge carrier mobility, adding an additional layer of responsiveness to the organic electronics. The group of Diao has been studying organic field effect transistors (OFETs) based on smart molecular crystalline switches of organic compounds such as DTBTBT,^[2]^ pentacene derivatives,[^2^, ^29^] and two‐dimensional quinoidal terthiophene (2DQTT) derivatives.^[80]^ In general, the charge carrier mobility is positively or negatively temperature‐dependent for a band‐like or hopping mechanism, respectively. The hole mobility of the HTS and LTS of DTBTBT has different sensitivity toward temperature changes, and the hole mobility drop becomes much faster after transition from LTS to HTS (Figure 8d).^[2]^ Consequently, the hole mobility of an OFET device based on DTBTBT can be switched by more than 200% when jumping between 318 and 363 K (would be around 15% without the phase transition; Figure 8d). The process appears to be fairly reversible. On the other hand, the hole mobility of 7,14‐bis[(trimethylsilyl)ethynyl]pentacene (TMSEP) also shows different temperature dependence for its LTS and HTS, albeit positively.^[2]^ However, because of the increase in contact resistance as a result of the higher temperature required for inducing phase transition, the OFET device based on TMSEP is less reversible during temperature change (Figure 8e). Note that TMSEP can also undergo four modes of SE or FE transitions. The large strain‐tolerance during the FE and SE transition is leveraged for an ultra‐flexible OFET device.^[29]^ As can be seen in Figure 8e, compared to other OFET devices based on organic molecules without FE or SE transition, the device based on TMSEP can withstand up to 13% strain without a detrimental effect on its performance. Furthermore, in a smaller strain range, the OFET device exhibits steady performance over several deformation cycles (Figure 8e). In addition to the p‐type organic semiconductors, the group of Diao also studied 2DQTT derivatives, a high‐performance non‐fullerene, small molecule, n‐type organic semiconductor.^[81]^ Recently, 2DQTT has been described as a crystalline switch; however, during phase transition the crystal is prone to fracturing due to substrate‐crystal adhesion.^[80]^ Interestingly, when transferring from LTS to HTS, 2DQTT shrinks along its long side, similar as a naphthalene diimide derivative reported previously.^[70]^ Such rare shrinkage upon heating is also applied as a key mechanism in an on‐off switching of electric circuits, but no metal coating for 2DQTT is needed because of its semiconductive nature. Besides, 2DQTT undergoes an irreversible slow non‐martensitic transition when heated up to 496 K due to thermally induced dimerization triggered by a higher diradical characteristic at high temperature. The shape of the crystal was roughly kept after the transition.

### 3.5 Tunable porous material

Sometimes, the molecular packing in the molecular crystal is not very compact, due to a number of reasons, and voids may exist in the crystal. For crystals of rigid molecules, such voids can be large enough to accommodate gases or other, even bigger molecules. It can be envisaged that when crystal undergoes a phase transition, the void and/or channels that are connected by void could be altered, forming a switchable molecular crystal shutter. Takasaki and Takamizawa demonstrated this concept with a supramolecular polymer crystal, Cu(II)_2_(benzoate)_4_(pyrazine).^[82]^ A single crystal of this material undergoes stress‐induced SE twinning, during which a rare 75.4°‐rotation of the crystal lattice from the parent to the product phase occurs. The rotation of the crystal lattice converts the channels that were once close‐to‐perpendicular to the direction of the stress, parallel to the stress. Consequently, the single crystal exhibits a high gas permission difference along certain crystallographic directions with and without shear stress‐induced twinning. For larger volume gases one could also expect an on‐off switching.

## 4 CONCLUSIONS

This review provides a comprehensive discussion of the dynamic field of smart molecular crystal switches, emphasizing the critical role of the molecular switches in the modern scientific and technological advancements. The potential of the molecular switches extends far beyond the currently available applications. Their integration into nanotechnology, smart materials, and medical applications promises to usher a new era of innovation. The continued exploration and development of these switches are crucial for unlocking their full potential, potentially leading to breakthroughs in targeted drug delivery systems, advanced material design, and nanoscale engineering.

Smart molecular crystal switches are facing challenges to harness their full potential for practical applications. While these switches exhibit remarkable properties, optimizing their performance across diverse conditions remains a complex task. Achieving precise control, scalability, switching efficiency, and environmental stability is essential for real‐life applications and integration into existing technologies. Researchers face the challenge of fine‐tuning these molecular systems to unlock their vast capabilities, presenting an ongoing quest for innovative solutions in the field of smart molecular crystal switches.

In summary, the realm of smart molecular crystal switches offers a rich landscape for scientific exploration and technological innovation. As we continue to unravel the intricacies of these systems, their profound impact on the future developments across various disciplines remains a promising prospect.

## CONFLICT OF INTEREST STATEMENT

The authors declare no conflicts of interest.

## Data Availability

The data that support the findings of this study are available on request from the corresponding author. The data are not publicly available due to privacy or ethical restrictions.
